# Supplementary material for: On the Effect of Bilateral Eye Movements on Memory Retrieval in Ageing and Dementia
Source: Brain Sci. 2022 Sep 27;12(10):1299. doi: 10.3390/brainsci12101299 (PMC9599909; doi:10.3390/brainsci12101299)
Supplement: Supplementary file 1 [file brainsci-12-01299-s001.zip › brainsci-1912907-supplementary.pdf]

Supplementary Materials

Experiment 1 – Word list 1

Target Words	Foil words
Evidence	Industry
Ceremony	Series
Patent	Volume
Miracle	Research
Session	Origin
Oxygen	Folly
Product	Answer
Master	Mischief
Conquest	Silence
Disease	Instance
Errand	Quality
Marriage	Excuse
Safety	Pleasure
Disaster	Devil
Maker	Colony
Property	Salary
Odour	Heaven
Capacity	Vapour
Injury	Opinion
Coffee	Agony
Trouble	Salute
Decree	Circuit
Assault	Poetry
Event	Fortune
Circuit	Tragedy
Owner	Array

Tribute	Effort
Method	Contents
Contract	History
Amount	Season
Revolt	Custom
Vision	Occasion
Quantity	Pressure
Poverty	Welfare
Ability	Advice
Comrade	Lecture

Experiment 2 – Additional word list 2

**Target Words**	**Foil words**
Diamond	Clothing
Ticket	Cellar
Captive	Cattle
Nephew	Abode
Dreamer	Temple
Hospital	Insect
Majority	Charter
Sunburn	Palace
Hearing	Fabric
Factory	Peach
Musician	Bottle
Twilight	Jelly
Sauce	Alcohol
Locker	Stain
Maiden	Portal
Hostage	Shock
Apple	Baron

Trunk
Meadow
Engine
Forehead
Toast
Bullet
Avenue
Fatigue
Hound
Garments
Painter
Kettle
Author
Snake
Tower
Metal
Edition
Costume
Clock

Goddess
Sickness
Sunset
Flesh
Nursery
Thief
Hotel
Bacteria
Settler
Cottage
Impact
Poster
Wheat
Rattle
Mileage
Pudding
Spray
Bloom
Robber
